# Interdisciplinary Team for MEDSReM-2 and Decision Support Through Pharmacology of Aging Principles

**DOI:** 10.1093/geroni/igab046.881

**Published:** 2021-12-17

**Authors:** Jeannie Lee, Kathleen Insel, J Nicholas, Amani Albadawi

**Affiliations:** 1 University of Arizona, Tucson, Arizona, United States; 2 University of Arizona College of Pharmacy, Tucson, Arizona, United States

## Abstract

The interdisciplinary team members with distinct and complementary expertise working collaboratively to advance MEDSReM to MEDSReM-2 will be introduced. The decision support functionality in MEDSReM-2 application (app) is to guide older users on making decisions about missed doses. MEDSReM-2 medication formulary was created to include safe hypertension medications for older adults. Pharmacology of aging, including Pharmacokinetic and Pharmacodynamic principles, along with published studies and expert peer reviews, were used to create an algorithm for safe window of time to take the missed medications. We will present the processes for developing the decision support algorithm for the MEDSReM-2 App and how this guide will be communicated to the users to inform their decision making about missed doses. Interdisciplinary collaboration including pharmacy, nursing, cognitive aging, and technology development that was crucial for designing and implementing decision support within the MEDSReM-2 app for older users will be shared.

